# Supplementary material for: Visual functions in children with craniopharyngioma at diagnosis: A systematic review
Source: PLoS One. 2020 Oct 1;15(10):e0240016. doi: 10.1371/journal.pone.0240016 (PMC7529266; doi:10.1371/journal.pone.0240016)
Supplement: S1 Appendix — (DOCX) [file pone.0240016.s001.docx]

**S1 Appendix. Search strategies for electronic databases**

**Search strategy for the Cochrane Library**

((("amblyopia":ti,ab OR lazy eye*:ti,ab OR "binocular vision":ti,ab OR "strabismus":ti,ab OR eye movement*:ti,ab OR "diplopia":ti,ab OR "double vision":ti,ab OR orthoptic*:ti,ab OR hemianop*:ti,ab OR "ocular":ti,ab OR vision*:ti,ab OR visual*:ti,ab OR ophthal*:ti,ab OR oculo*:ti,ab OR optic*:ti,ab OR optic chiasm*:ti,ab OR chiasma optic*:ti,ab OR optic decussation*:ti,ab OR optic compression*:ti,ab OR chiasm compression*:ti,ab OR chiasmal compression*:ti,ab OR OCT:ti,ab OR “optic coherence tomography”:ti,ab OR “optical coherence tomography”:ti,ab OR RNFL:ti,ab OR “GCL-IPL”:ti,ab OR retinal nerve fiber layer*:ti,ab OR ganglion cell layer*:ti,ab OR retinal layer*:ti,ab) OR (MeSH descriptor: [Amblyopia] OR MeSH descriptor: [Strabmismus] OR MeSH descriptor: [Vision, Binocular] OR MeSH descriptor: [Eye Movements] OR MeSH descriptor: [Diplopia] OR MeSH descriptor: [Orthoptics] OR MeSH descriptor: Tomography, Optical Coherence OR MeSH descriptor: [Optic Chiasm] OR MeSH descriptor: [Visual acuity] OR MeSH descriptor: [Visual Fields] OR MeSH descriptor: [Vision, Ocular] OR MeSH descriptor: [Orthoptics] OR MeSH descriptor: [Hemianopsia])) AND (MeSH descriptor: [Craniopharyngioma] OR craniopharyng*:ti,ab))

**Search strategy for Embase database**

(((‘amblyopia’:ti,ab,kw OR ‘lazy eye*’:ti,ab,kw OR ‘binocular vision’:ti,ab,kw OR ‘strabismus’:ti,ab,kw OR ‘eye movement*’:ti,ab,kw OR ‘diplopia’:ti,ab,kw OR ‘double vision’:ti,ab,kw OR ‘orthoptic*’:ti,ab,kw OR ‘hemianop*’:ti,ab,kw OR ‘ocular’:ti,ab,kw OR ‘vision*’:ti,ab,kw OR ‘visual*’:ti,ab,kw OR ‘ophthal*’:ti,ab,kw OR ‘oculo*’:ti,ab,kw OR ‘optic*’:ti,ab,kw OR ‘optic chiasm*’:ti,ab,kw OR ‘chiasma optic*’:ti,ab,kw OR ‘optic decussation*’:ti,ab,kw OR ‘optic compression*’:ti,ab,kw OR ‘chiasm compression*’:ti,ab,kw OR ‘chiasmal compression*’:ti,ab,kw OR ‘OCT’:ti,ab,kw OR ‘optic coherence tomography’:ti,ab,kw OR ‘optical coherence tomography’:ti,ab,kw OR ‘RNFL’:ti,ab,kw OR ‘GCL-IPL’:ti,ab,kw OR ‘retinal nerve fiber layer*’:ti,ab,kw OR ‘ganglion cell layer*’:ti,ab,kw OR ‘retinal layer*’:ti,ab,kw) OR (‘Amblyopia’/exp OR ‘Strabismus’/exp OR ‘Binocular Vision’/ex OR ‘Eye Movement’/exp OR ‘Diplopia’/exp OR ‘Orthoptics’/exp OR ‘Optical Coherence Tomography’/exp OR ‘Optic Chiasm’/exp OR ‘Visual Acuity’/exp OR ‘Visual Field’/exp OR ‘Vision’/exp OR ‘Orthoptics’/exp OR ‘Hemianopia’/exp)) AND (‘craniopharyngioma’/exp OR ‘craniopharyng*’:ti,ab,kw))

**Search strategy for PubMed database**

(((((amblyopia[tiab] OR lazy eye*[tiab] OR strabismus[tiab] OR “binocular vision”[tiab] OR eye movement*[tiab] OR diplopia[tiab] OR “double vision”[tiab] OR orthoptic*[tiab] OR hemianop*[tiab] OR ocular[tiab] OR vision*[tiab] OR visual*[tiab] OR ophthal*[tiab] OR oculo*[tiab] OR optic*[tiab] OR optic chiasm*[tiab] OR chiasma optic*[tiab] OR optic decussation*[tiab] OR optic compression*[tiab] OR chiasm compression*[tiab] OR chiasmal compression*[tiab] OR OCT[tiab] OR “optic coherence tomography” [tiab] OR “optical coherence tomography”[tiab] OR RNFL[tiab] OR “GCL-IPL” [tiab] OR retinal nerve fiber layer*[tiab] OR ganglion cell layer*[tiab] OR retinal layer*[tiab]))) OR (("Amblyopia"[Mesh] OR "Strabismus"[Mesh] OR "Vision, Binocular"[Mesh] OR "Eye Movements"[Mesh] OR "Diplopia"[Mesh] OR "Orthoptics"[Mesh] OR "vision, ocular"[Mesh] OR "visual fields"[Mesh] OR "Tomography, Optical Coherence"[Mesh] OR "Optic Chiasm"[Mesh] OR "visual acuity"[Mesh] OR "visual fields"[Mesh])))) AND (("craniopharyngioma"[Mesh] OR craniopharyng*[tiab]))
