# Supplementary material for: Visual functions in children with craniopharyngioma at diagnosis: A systematic review
Source: PLoS One. 2020 Oct 1;15(10):e0240016. doi: 10.1371/journal.pone.0240016 (PMC7529266; doi:10.1371/journal.pone.0240016)
Supplement: S3 Appendix — (PDF) [file pone.0240016.s003.pdf]

Systematic review

1. * Review title.

Give the working title of the review, for example the one used for obtaining funding. Ideally the title should state succinctly the interventions or exposures being reviewed and the associated health or social problems. Where appropriate, the title should use the PI(E)COS structure to contain information on the Participants, Intervention (or Exposure) and Comparison groups, the Outcomes to be measured and Study designs to be included.

Visual function in children with craniopharyngioma at diagnosis: a systematic review

2. Original language title.

For reviews in languages other than English, this field should be used to enter the title in the language of the review. This will be displayed together with the English language title.

3. * Anticipated or actual start date.

Give the date when the systematic review commenced, or is expected to commence.

30/09/2019

4. * Anticipated completion date.

Give the date by which the review is expected to be completed.

22/04/2020

5. * Stage of review at time of this submission.

Indicate the stage of progress of the review by ticking the relevant Started and Completed boxes. Additional information may be added in the free text box provided.
Please note: Reviews that have progressed beyond the point of completing data extraction at the time of initial registration are not eligible for inclusion in PROSPERO. Should evidence of incorrect status and/or completion date being supplied at the time of submission come to light, the content of the PROSPERO record will be removed leaving only the title and named contact details and a statement that inaccuracies in the stage of the review date had been identified.
This field should be updated when any amendments are made to a published record and on completion and publication of the review. If this field was pre-populated from the initial screening questions then you are not able to edit it until the record is published.

The review has not yet started: No

Review stage	Started	Completed
Preliminary searches	Yes	No
Piloting of the study selection process	No	No
Formal screening of search results against eligibility criteria	No	No
Data extraction	No	No
Risk of bias (quality) assessment	No	No
Data analysis	No	No

Provide any other relevant information about the stage of the review here (e.g. Funded proposal, protocol not yet finalised).

6. * Named contact.

The named contact acts as the guarantor for the accuracy of the information presented in the register record.

Myrthe Nuijts

Email salutation (e.g. "Dr Smith" or "Joanne") for correspondence:

M. Nuijts

7. * Named contact email.

Give the electronic mail address of the named contact.

M.A.Nuijts@umcutrecht.nl

8. Named contact address

Give the full postal address for the named contact.

UMC Utrecht, Heidelberglaan 100, 3508 GA Utrecht, post box 85500, NL

9. Named contact phone number.

Give the telephone number for the named contact, including international dialling code.

10. * Organisational affiliation of the review.

Full title of the organisational affiliations for this review and website address if available. This field may be completed as 'None' if the review is not affiliated to any organisation.

Universitair Medisch Centrum Utrecht

Organisation web address:

www.umcutrecht.nl

11. * Review team members and their organisational affiliations.

Give the personal details and the organisational affiliations of each member of the review team. Affiliation

refers to groups or organisations to which review team members belong. **NOTE: email and country are now mandatory fields for each person.**

Miss Nienke Veldhuis. Utrecht University
Miss M. Nuijts. Universitair Medisch Centrum Utrecht
Dr I. Stegeman. Universitair Medisch Centrum Utrecht
Dr A.Y. N. Schouten-van Meeteren. Prinses Máxima Centrum
Professor S.M. Imhof. Universitair Medisch Centrum Utrecht

12. * Funding sources/sponsors.

Give details of the individuals, organizations, groups or other legal entities who take responsibility for initiating, managing, sponsoring and/or financing the review. Include any unique identification numbers assigned to the review by the individuals or bodies listed.

This systematic review is initiated by Dr. A.Y.N. Schouten-van Meeteren and prof. Dr. S.M. Imhof for an internship of Miss N. Veldhuis, medical student at Utrecht University, in the context of the CCISS study. Dr. I. Stegeman works as an epidemiologist at UMC Utrecht and is the main supervisor of N. Veldhuis during her internship and writing of the systematic review. Drs. M. Nuijts is a PhD candidate of the CCISS study and will take the daily supervision of N. Veldhuis for her responsibility.

Grant number(s)

13. * Conflicts of interest.

List any conditions that could lead to actual or perceived undue influence on judgements concerning the main topic investigated in the review.

None

14. Collaborators.

Give the name and affiliation of any individuals or organisations who are working on the review but who are not listed as review team members. **NOTE: email and country are now mandatory fields for each person.**

15. * Review question.

State the question(s) to be addressed by the review, clearly and precisely. Review questions may be specific or broad. It may be appropriate to break very broad questions down into a series of related more specific questions. Questions may be framed or refined using PI(E)COS where relevant.

What is the visual function in children with craniopharyngioma at diagnosis and at 1-, 3- and 5-year follow-up, considering visual acuity, visual fields, fundoscopy and orthoptic examination?

16. * Searches.

State the sources that will be searched. Give the search dates, and any restrictions (e.g. language or publication period). Do NOT enter the full search strategy (it may be provided as a link or attachment.)

After finishing our search strategy, we will search in PubMed, Embase and The Cochrane Library.
Our systematic review will contain articles until the 1st of October 2019.

There will be no restrictions in our search.

17. URL to search strategy.

Give a link to a published pdf/word document detailing either the search strategy or an example of a search

strategy for a specific database if available (including the keywords that will be used in the search strategies), or upload your search strategy.Do NOT provide links to your search results.

Alternatively, upload your search strategy to CRD in pdf format. Please note that by doing so you are consenting to the file being made publicly accessible.

Do not make this file publicly available until the review is complete

18. * Condition or domain being studied.

Give a short description of the disease, condition or healthcare domain being studied. This could include health and wellbeing outcomes.

We will study visual function in children with craniopharyngiomas. Craniopharyngiomas are located nearby important visual structures in the brain, therefore this population is at risk of impaired visual function.

19. * Participants/population.

Give summary criteria for the participants or populations being studied by the review. The preferred format includes details of both inclusion and exclusion criteria.

Inclusion criteria: Children aged 0 to 18 years with craniopharyngiomas, who have undergone ophthalmic examination.

Exclusion criteria: Patients above 18 years with craniopharyngiomas.

20. * Intervention(s), exposure(s).

Give full and clear descriptions or definitions of the nature of the interventions or the exposures to be reviewed.

In our systematic review we will describe the visual function, considering visual acuity, visual fields, fundoscopy and orthoptic testing, in children with craniopharyngioma at diagnosis and during 1-, 3- and 5-year follow-up.

21. * Comparator(s)/control.

Where relevant, give details of the alternatives against which the main subject/topic of the review will be compared (e.g. another intervention or a non-exposed control group). The preferred format includes details of both inclusion and exclusion criteria.

Not applicable.

22. * Types of study to be included.

Give details of the types of study (study designs) eligible for inclusion in the review. If there are no restrictions on the types of study design eligible for inclusion, or certain study types are excluded, this should be stated. The preferred format includes details of both inclusion and exclusion criteria.

We will include cohort studies and case series. Reviews will not be included.

23. Context.

Give summary details of the setting and other relevant characteristics which help define the inclusion or exclusion criteria.

Only studies in which visual function in children (0-18 years) with craniopharyngiomas is addressed will be included.

24. * Main outcome(s).

Give the pre-specified main (most important) outcomes of the review, including details of how the outcome is defined and measured and when these measurement are made, if these are part of the review inclusion criteria.

The purpose of our systematic review is to give an accurate overview of current available studies about visual function in children with craniopharyngiomas. In more detail the main outcomes of the review are results from visual field, visual acuity, fundoscopy and orthoptic examination in children with craniopharyngioma.

* Measures of effect

Please specify the effect measure(s) for you main outcome(s) e.g. relative risks, odds ratios, risk difference, and/or 'number needed to treat.

At baseline, 1-, 3- and 5-year follow-up.

25. * Additional outcome(s).

List the pre-specified additional outcomes of the review, with a similar level of detail to that required for main outcomes. Where there are no additional outcomes please state 'None' or 'Not applicable' as appropriate to the review

None

* Measures of effect

Please specify the effect measure(s) for you additional outcome(s) e.g. relative risks, odds ratios, risk difference, and/or 'number needed to treat.

Not applicable.

26. * Data extraction (selection and coding).

Describe how studies will be selected for inclusion. State what data will be extracted or obtained. State how this will be done and recorded.

Study selection:
Two review authors will independently select studies for inclusion in our systematic review. They will first screen for eligibility by reading title/abstracts and afterwards by reading full text of these potentially eligible studies.

The reviewers will be blinded to each other's decisions. The two reviewers are going to compare the selected studies with each other. In case of discrepancy between the selected studies the reviewers will discuss whether the study should be included or not. Rayyan will be used for recording decisions.

Data extraction:

Extracted data will include: study characteristics (for instance, study author); participant demographics and baseline characteristics (for instance, age); measurements (visual acuity, visual field, fundoscopy and orthoptic testing) at diagnosis and follow-up (1, 3, 5 years); information for assessment of the risk of bias.

Two reviewers will extract data received data from included studies independently. In case of disagreement

between the two reviewer authors about the extracted data, they will discuss this together - with help of a third author where necessary - in order to come to an agreement. The reviewers will screen references of selected studies whether there are other studies worth including that are not included yet. Missing data will be requested from study authors by mail. Data from included articles will be recorded in an Excel spreadsheet and Word document. Rayyan will be used for the screening and selection of titles and abstracts and afterwards for the data extraction.

The extracted data will be about study aims, study design and setting, number of children, tumour subtype, age at diagnosis, gender, tumour location, visual impairment, vision defects (including visual acuity, visual fields, orthoptics, fundoscopy) and ophthalmic examination.

27. * Risk of bias (quality) assessment.

Describe the method of assessing risk of bias or quality assessment. State which characteristics of the studies will be assessed and any formal risk of bias tools that will be used.

We will use the NOS (Newcastle-Ottawa Scale) for assessing the risk of bias of the included studies. We will assess the risk of bias of studies based on three domains for each study individually, including selection, comparability and outcome. For each domain, stars will be allocated based on predefined criteria.

28. * Strategy for data synthesis.

Provide details of the planned synthesis including a rationale for the methods selected. This **must not be generic text** but should be **specific to your review** and describe how the proposed analysis will be applied to your data.

We will include a minimum number of ten studies in our systematic review. Of studies with overlapping inclusion periods and hospitals, we will exclude studies with the shortest period of patient inclusion and/or the least availability of ophthalmological data for final data extraction due to the possibility of overlapping study samples.

We will extract data about children with primary craniopharyngioma.

We will present the extracted data in two tables, one table about general characteristics of the included studies and one table about visual function in children with craniopharyngioma at diagnosis. The data will be quantified per item (tumour location, visual impairment, visual acuity, visual fields, orthoptics, fundoscopy and other vision related defects) and we will present numbers for subtypes of each item together with an percentage in our review. No statistic models will be used for this systematic review. To assess the quality of included studies we will use the Newcastle Ottawa Scale (NOS), no studies will be excluded based on having a high risk of bias. Due to variations between studies, we are not able to perform a meta-analysis.

29. * Analysis of subgroups or subsets.

State any planned investigation of ‘subgroups’. Be clear and specific about which type of study or participant will be included in each group or covariate investigated. State the planned analytic approach.

If the necessary data are available, subgroup analyses will be done for patients diagnosed with

craniopharyngiomas with different lengths of time between onset of symptoms and diagnosis and if necessary subgroup analysis by age.

30. * Type and method of review.

Select the type of review and the review method from the lists below. Select the health area(s) of interest for your review.

Type of review

Cost effectiveness
No

Diagnostic
No

Epidemiologic
No

Individual patient data (IPD) meta-analysis
No

Intervention
No

Meta-analysis
No

Methodology
No

Narrative synthesis
Yes

Network meta-analysis
No

Pre-clinical
No

Prevention
No

Prognostic
No

Prospective meta-analysis (PMA)
No

Review of reviews
No

Service delivery
No

Synthesis of qualitative studies
No

Systematic review
Yes

Other
No

Health area of the review

Alcohol/substance misuse/abuse
No

Blood and immune system

No

Cancer
Yes

Cardiovascular
No

Care of the elderly
No

Child health
Yes

Complementary therapies
No

COVID-19
No

Crime and justice
No

Dental
No

Digestive system
No

Ear, nose and throat
No

Education
No

Endocrine and metabolic disorders
No

Eye disorders
Yes

General interest
No

Genetics
No

Health inequalities/health equity
No

Infections and infestations
No

International development
No

Mental health and behavioural conditions
No

Musculoskeletal
No

Neurological
Yes

Nursing
No

Obstetrics and gynaecology
No

Oral health
No

Palliative care
No

Perioperative care
No

Physiotherapy
No

Pregnancy and childbirth
No

Public health (including social determinants of health)
No

Rehabilitation
No

Respiratory disorders
No

Service delivery
No

Skin disorders
No

Social care
No

Surgery
No

Tropical Medicine
No

Urological
No

Wounds, injuries and accidents
No

Violence and abuse
No

31. Language.

Select each language individually to add it to the list below, use the bin icon to remove any added in error.
English

There is not an English language summary

32. * Country.

Select the country in which the review is being carried out from the drop down list. For multi-national collaborations select all the countries involved.

Netherlands

33. Other registration details.

Give the name of any organisation where the systematic review title or protocol is registered (such as with The Campbell Collaboration, or The Joanna Briggs Institute) together with any unique identification number assigned. (N.B. Registration details for Cochrane protocols will be automatically entered). If extracted data will be stored and made available through a repository such as the Systematic Review Data Repository (SRDR), details and a link should be included here. If none, leave blank.

34. Reference and/or URL for published protocol.

Give the citation and link for the published protocol, if there is one

Give the link to the published protocol.

Alternatively, upload your published protocol to CRD in pdf format. Please note that by doing so you are consenting to the file being made publicly accessible.

No I do not make this file publicly available until the review is complete

Please note that the information required in the PROSPERO registration form must be completed in full even if access to a protocol is given.

35. Dissemination plans.

Give brief details of plans for communicating essential messages from the review to the appropriate audiences.

After finishing our systematic review we intend to publish it in a journal with relevance to the subject.

Do you intend to publish the review on completion?

Yes

36. Keywords.

Give words or phrases that best describe the review. Separate keywords with a semicolon or new line. Keywords will help users find the review in the Register (the words do not appear in the public record but are included in searches). Be as specific and precise as possible. Avoid acronyms and abbreviations unless these are in wide use.

Systematic review; brain tumor; craniopharyngioma; children; visual function; optic chiasm.

37. Details of any existing review of the same topic by the same authors.

Give details of earlier versions of the systematic review if an update of an existing review is being registered, including full bibliographic reference if possible.

38. * Current review status.

Review status should be updated when the review is completed and when it is published. For newregistrations the review must be Ongoing.
Please provide anticipated publication date

Review_Ongoing

39. Any additional information.

Provide any other information the review team feel is relevant to the registration of the review.

After the first assessment of Prospero on 13-03-2020 and during the writing process of our systematic review we made the following amendments in our registration form:

#1, #15, #20 and #24: We decided to extract data about fundoscopy as well as data about visual acuity, visual fields and orthoptic examination as we had initially planned.

#3: The anticipated completion date was changed from 20-12-2019 to 22-04-2020. The process of writing this systematic review took more time than we had expected due to the high number of studies that were eligible for inclusion.

#27: We decided to use the NOS (Newcastle-Ottawa scale) for assessing the risk of bias of studies included instead of the QUIPS (quality in prognostic studies) tool as we had initially planned, because we found out

that the NOS was more suitable for the design of our systematic review than the QUIPS tool.

#28: We made some amendments to field #28 after the first assessment of Prospero. Instead of a generic statement about our strategy for data synthesis, we have changed this field in order to provide more concrete details about our strategy for data synthesis.

40. Details of final report/publication(s).

This field should be left empty until details of the completed review are available.

Give the link to the published review.
