# Supplementary material for: Visual functions in children with craniopharyngioma at diagnosis: A systematic review
Source: PLoS One. 2020 Oct 1;15(10):e0240016. doi: 10.1371/journal.pone.0240016 (PMC7529266; doi:10.1371/journal.pone.0240016)
Supplement: Attachment — Submitted filename: Reponse to Reviewers.docx [file pone.0240016.s004.docx]

Myrthe Nuijts, MD

University Medical Center Utrecht

PO Box 85500

NL-3508 GA Utrecht

Phone: +31 88 75 71305

Fax: +31 88 75 55417

Email: M.A.Nuijts@umcutrecht.nl

Utrecht, September 7, 2020

Dear Professor Awadein,

Please find attached our revised submission titled ‘’Visual functions in children with craniopharyngioma at diagnosis: a systematic review’’, which we would like you to consider for publication in *PLOS ONE*.

We thank the editor and reviewers for their careful consideration, and are pleased to have had the opportunity to revise our manuscript. Please find below a point-by-point response to all comments.

We much appreciate the effort taken to reevaluate our manuscript and look forward to your reply.

Sincerely on behalf of all authors,

Myrthe Nuijts, MD

Corresponding author

|  | **Reviewer 1** | **Response** | **Manuscript changes** |
| --- | --- | --- | --- |
| 1 | I suggest that the full title is modified to "Visual functions at diagnosis in children with craniopharyngioma: a systematic review". | We thank the reviewer for this comment. We agree with the suggested full title. | Page 1, line 4 |
| 2 | The running title needs also to be modified to "Visual functions in childhood craniopharyngioma". | We agree with the suggested running title. | Page 1, line 7 |
| 3 | Page 9, lines 198-199: this sentence needs re-phrasing to "As regards to the included children with CP, 1236 were females and the mean age ranged from 0 to 23 years". | In the revised manuscript, we rephrased this sentence to ‘’With regard to the included children with CP, 1236 were females and the mean age ranged from 0 to 23 years’’. | Page 8, lines 183-185. |
| 4 | Page 9, lines 209-210: this sentence needs re-phrasing to "Precisely only 10 studies reported primarily visual function and/or long term visual outcomes in children with CP". | In the revised manuscript, we rephrased this sentence. | Page 9, lines 195-196. |
| 5 | In pages 28-29, lines 289-295: the term visual impairment is vague, could the authors clarify what do they exactly mean by visual impairment? if they mean visual acuity and visual field then this item should be not be mentioned separately. | We thank the reviewer for this comment and we agree that the term visual impairment is vague. Authors frequently reported about the total number of patients with visual impairment in general without describing results of visual acuity and visual field testing. Therefore, we decided to report this data alongside the results of visual acuity and visual field testing. We rephrased this sentence in the results section aiming to clarify what we mean by using the term ‘visual impairment’. | Page 28, lines 276-285. |
| 6 | In page 29, visual acuity paragraph: the authors mentioned that 53 studies out of 84 studies described visual acuity at diagnosis and only 4 studies referred to visual acuity as BCVA. Is there an explanation for that? How do the authors justify using UCVA and BCVA as a description for visual acuity affection in these children? | Unfortunately, only in seven of the included studies authors explicitly described that they used BCVA as a description for visual acuity affection. In the other studies, authors only reported ‘visual acuity’ without specifying whether they used BCVA or UCVA. In the revised manuscript, we added a sentence to clarify this and added UCVA to the list of abbreviations. | Page 29, lines 292-294.  Page 38, lines 511-512. |

|  | **Reviewer 2** | **Response** | **Manuscript changes** |
| --- | --- | --- | --- |
| 1 | Could the authors check the reviewed studies, which testing methods were used (and feasible) in different age groups. Perhaps these analyses could lead to a cautious recommendation of what should be done in terms of ophthalmological diagnostics at different age periods. | We thank the reviewer for this comment and agree that this is very important to reliably compare results of different studies. Table 3 of our manuscript shows the ophthalmologic testing methods that were used in the different studies (if provided by the authors). Regrettably, it shows that the majority of studies did not report the methods they used for ophthalmologic testing. Therefore, unfortunately we were not able to compare feasibility of testing methods for different age groups due to lacking data. This is outlined in the discussion section (see ‘limitations of the included studies’ and ‘recommendations’). | Page 36, lines 457 – 460.  Page 37, lines 493-497. |
